# A Path Model of the Relationship between Mood, Exercise Behavior, Coping, and Mental Health among Malaysians during the COVID-19 Pandemic

**DOI:** 10.3390/ijerph19105939

**Published:** 2022-05-13

**Authors:** Jing Mun Yew, Yee Cheng Kueh, Bachok Norsa’adah, Foo Weng Leong, Heen Yeong Tang, Garry Kuan

**Affiliations:** 1 Biostatistics and Research Methodology Unit, School of Medical Sciences, Universiti Sains Malaysia, Kubang Kerian 16150, Kelantan, Malaysia; daphneyew0930@gmail.com (J.M.Y.); norsaadah@usm.my (B.N.); 2 Department of Psychiatry, RCSI-UCD Malaysia Campus 4, Jalan Sepoy Lines, Georgetown 10450, Penang, Malaysia; lamblfw@gmail.com; 3 Access Hitech Automation Sdn. Bhd., Batu Maung 11960, Penang, Malaysia; hytang@accesshitech.com; 4 Exercise and Sports Science Programme, School of Health Sciences, Universiti Sains Malaysia, Kubang Kerian 16150, Kelantan, Malaysia

**Keywords:** COVID-19, structural equation modelling, psychology, stress, depression, anxiety

## Abstract

The purpose of this study was to examine the relationship between the mood, physical activity, coping, and mental health of Malaysians during the COVID-19 pandemic. A cross-sectional study was conducted via an online survey, with self-administered questionnaires. The respondents were recruited using snowball sampling techniques. The Brunel Mood Scale (BRUMS), the Exercise Regulations in Exercise-3 (BREQ-3), the Brief Coping Orientation of Problem Experienced (Brief COPE), and the Depression, Anxiety, and Stress Scale (DASS-21) were used. A path analysis was conducted on the data. A total of 842 people participated in the survey. The mean age of participants was 22 years (interquartile range = 6) and 24.0% were male. The final path model fitted the data well, with a comparative fit index of 0.998, a Tucker–Lewis index of 0.988, a standardized root mean square residual of 0.001, and a root mean square error of approximation of 0.072. In this study, there were significant path relationships between mood, exercise behavior, coping, and mental health. Additionally, it was demonstrated that the variables of mood, exercise behavior, and coping have both direct and indirect effects on mental health. The results also suggested that utilizing appropriate coping skills, exercise behavior, and positive mood can directly lower levels of depression, anxiety and stress, and that appropriate coping skills and positive mood can directly affect exercise behavior.

## 1. Introduction

In late December 2019, Wuhan, Hubei Province, China reported numerous unexplained medical conditions, including pneumonia. Wuhan was the first city in China to report coronavirus cases, as the virus spread rapidly throughout the world, posing a serious threat to global health [1]. Exposure to a Wuhan seafood market was implicated in the majority of cases [1]. The World Health Organization (WHO) designated this outbreak as Coronavirus Disease 2019 (COVID-19) on 11 February 2020 [2]. The WHO declared COVID-19 a pandemic in March 2020 due to the increasing number of cases in over 200 countries and territories. As a result, the Malaysian government implemented a nationwide movement control order (MCO), with the goal of containing the outbreak. The MCO officially began on 18 March 2020. Throughout Malaysia, all offices, schools, universities, shops, and businesses were closed [3,4]. During this time period, the entire Malaysian population were required to work or study entirely from home, except for those providing essential services, including the following: energy and electricity, telecommunication, water, postal services, oil and gas, fuel, lubricants, broadcasting, finance and banking, health, firefighting, security and defense, transportation and logistics, cleaning, retail, and food [5]. Malaysians were only permitted to leave the house for essential activities, such as grocery shopping and medical treatment, during this time period in order to contain the COVID-19 outbreak.

### 1.1. Effect of Quarantine and Lockdown Measures

Individuals’ negative emotions were exacerbated by the closure of schools and businesses [6]. Isolation also had an effect on peoples’ moods and psychological responses [6,7]. While social distancing is a protective measure against virus attacks transmitted via contact, social distancing is a psychological and social problem in and of itself, with the potential to exacerbate mental health problems, such as depression and anxiety, and to disrupt sleep [6,8]. Additionally, quarantine and lockdown measures can also erode human relationships and alter individuals’ behaviors and lifestyles, such as their ability to maintain physical activity while isolating [9]. An extended time spent at home (in so-called “quarantine”) can be associated with sedentary behaviors (sitting, watching television, or using smart devices), insufficient physical activity, and engaging in avoidance behaviors, all of which can exacerbate or increase the risk of chronic health conditions [10]. Having enough sleep can also lead to a healthier lifestyle. However, during one study it was found that there were both positive and negative associations between school closures and adolescents’ health. The study showed a negative association between school closures and adolescents’ health through the psychological distress caused, but a beneficial association between them through the increase in their sleep duration [11]. Another investigation found that COVID-19 had no impact on Iranian Adults’ attendance of team sporting activities during the confinement [12]. Therefore, the COVID-19 pandemic had both a bright and a dark side for society: while it taught people ways to overcome distress, it also increased peoples’ experience of psychological distress [13].

### 1.2. Impact of COVID-19 Pandemic and Its Investigation

Fear and anxiety have been caused by the COVID-19 pandemic, and there is an urgent need to understand the mental health status of outbreak communities. The mental health issues associated with the COVID-19 pandemic, as well as the appropriate behaviors that people can use to avoid the mental health threats associated with the infection, have rarely been investigated promptly. Additionally, it is unknown whether coping behaviors were enhanced in the outbreak populations as a means of coping with the crisis [9]. More pragmatic responses, such as physical activity, may be used to cope with negative emotions during a new pandemic crisis. Adaptive coping skills, as well as exercise behavior, were associated with good mental health [8]. Additionally, there was a correlation between an individual’s mood, exercise behavior, and coping ability [14]. However, few studies have examined this association during the outbreak of a disease such as COVID-19.

**Figure 1:**
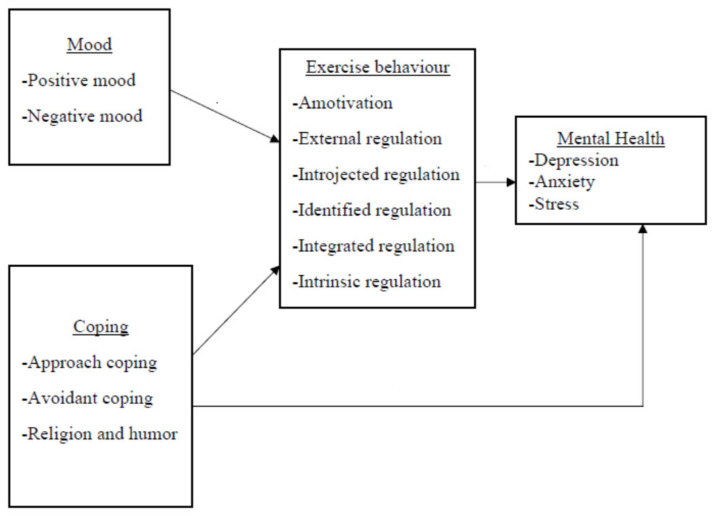
Proposed hypothesized relationships of mood state, coping skills, exercise behavior, and mental health.

There is still a dearth of information regarding the psychological effects of COVID-19 on Malaysians. Additionally, there is a dearth of information available to the general public on how to cope with the COVID-19 outbreak. Instead, moods are a complex combination of emotions that influence exercise behavior [15]. This study may help us better understand how the public responds to pandemic outbreaks. We hoped to gain a better understanding of the interplay between mood, exercise behavior, coping, and mental health in the Malaysian population during the COVID-19 pandemic (see Figure 1). The purpose of this study was to ascertain the direct and indirect path relationships between the study variables.

## 2. Materials and Methods

### 2.1. Participants

An online cross-sectional study was conducted, with 842 participants. Data were collected between 23 April 2020 and 17 July 2020. The snowball sampling method was used. Data were collected through WhatsApp, Facebook, and email. All Malaysians who met the eligibility criteria (being 18 years old or older, being able to read and understand Bahasa English and Bahasa Malaysia, and being available at the time of data collection) were invited to take part in the survey. Those who did not have internet access were excluded. 

### 2.2. Ethical Considerations

The Human Research Ethics Committee of the Universiti Sains Malaysia approved the study [USM/JEPeM/COVID19-11] and it was conducted in accordance with the Declaration of Helsinki. On the first page of the online survey form, participants were provided with research information. The research information included the study’s purpose, its procedures, the potential risks associated with participation, and the benefits associated with participation in this study. After this, each respondent provided their informed consent to participate in the study. All respondents volunteered to participate in this study.

### 2.3. Questionnaires

Sociodemographic data comprised the first section of the questionnaire. The following four questionnaires were also included: the Brunel Mood Scale (BRUMS); the Behavioral Regulation in Exercise Questionnaire (BREQ-3); the Brief Coping Orientation of Problem Experienced (Brief COPE); and the Depression, Anxiety and Stress Scale (DASS-21). These questionnaires were used to determine the mood, exercise behaviors, coping skills used, and the mental health status of the participants.

#### 2.3.1. Sociodemographic Data

The participants’ age, gender, ethnic origin, exercise frequency, exercise sessions, positive COVID-19 test results, state of residence in Malaysia, occupation, household income, and educational level were all included in the data collection.

#### 2.3.2. Brunel Mood Scale (BRUMS)

The Brunel Mood scale is used to gauge populations’ moods. The questionnaire contains 24 items and six subscales: anger, confusion, depression, vigor, fatigue, and tension [16]. The items were rated on a 5-point Likert scale, ranging from 0 (not at all) to 4 (extremely). The BRUMS categorizes moods into two categories: positive mood (vigor) and negative mood (anger, confusion, depression, fatigue, and tension). These two moods were compared in the study. The BRUMS’s English translation was found to be valid and reliable. A confirmatory factor analysis (CFA) revealed an acceptable fit, with a comparative fit index (CFI: 0.93), a root mean square error of approximation (RMSEA: 0.06), and a Cronbach alpha all greater than 0.70 [17]. BRUMS has been translated into Malay, with an acceptable Cronbach alpha of between 0.70 and 0.77 [18]. The Malay version of BRUMS demonstrated a high degree of correspondence with the CFA findings: CFI = 0.90 and RMSEA = 0.05 [18].

#### 2.3.3. Behavioral Regulation in Exercise (BREQ-3)

BREQ-3 evaluates an individual’s exercise behavior [19]. It is composed of 24 items and 6 subscales. Amotivation, external regulation, self-regulation, identified regulation, integrated regulation, and intrinsic regulation are the subscales. The responses were rated on a 5-point Likert scale, ranging from 0 (not true for me), to 2 (occasionally true for me), to 4 (very true for me). This indicated the importance of a reason in influencing an individual’s decision to engage in physical activity. According to reports, the English version of BREQ-3 was valid and reliable. CFI = 0.91; RMSEA = 0.06; SRMR = 0.06; and Cronbach alpha values ranged between 0.66 and 0.87. The BREQ-3 questionnaire was translated into Malay and administered to Malaysian citizens [20]. CFI = 0.949, TLI = 0.938, and RMSEA = 0.04, with composite reliability ranging from 0.746 to 0.841 for the Malay version of BREQ-3 [20].

#### 2.3.4. Brief Coping Orientation of Problem Experienced (Brief COPE)

The Brief Coping Orientation of Problem Experienced (Brief COPE) questionnaire [21] includes 28 items concerning how individuals have coped with their most stressful experience. It rates each item separately on the following scale: 1 (I have not been doing this at all), 2 (I have been doing this a little), 3 (I have been doing this a medium amount), and 4 (I have been doing this a lot). There are three main domains of coping strategies, which are as follows: approach strategies (a combination of active coping, using emotional support, using instrument support, positive reinterpretation, planning, and acceptance); avoidant strategies (a combination of self-distraction, denial, substance abuse, behavioral disengagement, a focus on and venting of emotions, and self-blame); and neither approach nor avoidant coping strategies (a combination of religion and humor). In the present study, these three coping strategies were used in the analysis. For the English version of Brief COPE, the fit indices were CFI = 0.94, TLI = 0.91, and RMSEA = 0.04 [21]. Brief COPE is a valid and reliable questionnaire in the Malay version, with a Cronbach alpha of 0.83 [22]. The Malay version of Brief COPE had been validated using EFA. The validity was found to be satisfactory, with a total variance extracted of 71.15% [22].

#### 2.3.5. Depression, Anxiety, and Stress Scale (DASS-21)

The Depression, Anxiety, and Stress Scale is a numerical rating scale for psychological distress [23]. It contains 21 items. The rating scale was as follows: 0 (did not apply to me at all—Never), 1 (applied to me to some extent, or some of the time—Occasionally), and 3 (applied to me much of the time, or most of the time—Almost always). The DASS score for each component of depression, anxiety, and stress can be expressed categorically or numerically. However, numerical scoring was used for each subscale in the current study’s data analysis. The English version was validated using exploratory structural equation modeling (ESEM) and was found to have an acceptable fit index (RMSEA = 0.056, SRMR = 0.055, TLI = 0.914, CFI = 0.926) and a satisfactory internal consistency (0.79–0.84) [24]. According to Nordin et al. [25], the Malay version of the DASS-21 is valid and reliable.

### 2.4. Sample Size Determination

The sample size of this study was determined based on the power of the hypotheses path model; a Monte Carlo simulation (Muthèn and Muthèn [26]), Mplus version 8.3, was used. The initial model was created using the syntax of Mplus. The estimated coefficient of path regression was 0.25. In this simulation, a different sample size was used, and the study’s power was calculated. A sample size of 800 is required to achieve a power of 0.82. 842 participants were recruited for this study.

### 2.5. Statistical Analyses

Preliminary data analysis was used to explore and clean up the data. IBM SPSS Statistics 27.0 was used to perform descriptive statistics. Mplus 8.3 was used to conduct the path analysis.

Prior to performing path analysis, item parceling was performed to accommodate the large number of items in a scale and to address the data’s non-normality [27]. Parceling is a term that refers to the process of adding two or more items from the original item scores to create parcel scores. Following that, these parcel scores are used as new measures of the underlying constructs [28]. If the parceled items are unidimensional, they are more likely than the original individual items to adhere to the multivariate normality assumptions [28]. Each of the study’s items (mood, exercise behavior, coping, and mental health) was subdivided into its own subscale. Following that, the path analysis model was used to test the relationships between the participants’ mood, exercise behavior, coping, and mental health.

The multivariate normality of skewness and kurtosis were determined using Mardia’s measure of multivariate normality. A *p*-value less than 0.05 indicates that the multivariate normality assumption has been violated, implying significant skewness and kurtosis. Mardia’s test for multivariate normality was not met in this study. As a result, the path analysis was performed using the robust maximum likelihood (MLR) estimator.

In the path analysis, the model fitness was determined using a variety of fit indices. The fit indices used were the comparative fit index (CFI) and a Tucker–Lewis index (TLI) of greater than 0.95, which indicate a good fit when combined [29]. After this, a standardized root mean square error (SRMR) of less than 0.08 indicates a good fit [30], while a root mean square approximation error (RMSEA) of less than 0.08 indicates a reasonably good fit [29,30]. If the model cannot adequately fit the data, it is re-specified. Model re-specification entails the elimination of insignificant pathways and problematic variables, as well as the addition of new pathways to the model. The finalized path model, which fitted the data well, was reported. The significant path coefficients (β) with 95% CI, standard error (SE), and statistical significance value were reported in a table. Statistical significance was defined as a *p*-value of 0.05.

## 3. Results

### 3.1. Participants’ Characteristics

The sociodemographic characteristics of the Malaysian respondents (*n* = 842) are shown in Table 1. The findings indicated that 202 (24.0%) of the participants were male and 640 (76.0%) were female. The respondents’ median age was 22 years; the youngest respondent was 19 years old, and the oldest was 66 years old. The majority (52.3%) of the respondents were Malay. A total of 72.2% of respondents indicated that they engaged in exercise sessions lasting longer than 10 min.

### 3.2. Path Model

As an initial model, Model 1, depicted in Figure 1, was tested. As shown in Table 2, the results of Model 1 indicated a poor fit of the data, based on CFI, TLI, and RMSEA. Model 1’s fit indices were insufficient and did not meet the recommended value (i.e., CFI, TLI, and RMSEA). The model needed to be re-specified by removing insignificant paths and those with a standardized estimate greater than one. Paths were removed one at a time and the model’s fitness was re-tested repeatedly. However, the researchers in this study only removed a pathway after adequate theoretical support was provided and discussed.

As shown in Table 2, Model 2 demonstrated the model fit indices after the non-significant path were removed, whereas Model 3 demonstrated the model fit indices after removing the problematic pathways with an estimated value greater than one. After removing non-significant paths, the results of the revised path Model 2 improved slightly. However, once the paths with a standardized estimate greater than one were eliminated, the revised Model 3 did not improve fitness as expected. The fit indices indicated a slight decline in the model fitness. Paths with a standardized estimate greater than one indicated the presence of some problems with multicollinearity between the two study variables connected by the path. The model was re-specified in light of the problematic variables identified.

**Figure 2:**
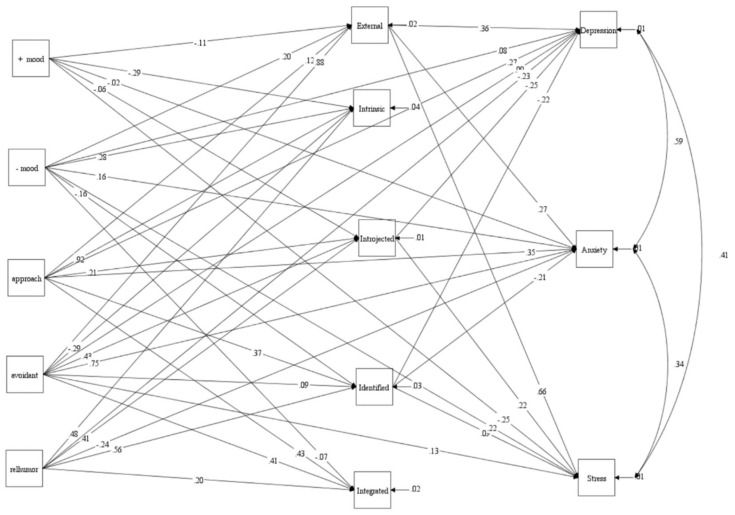
Path diagram of the final model (Model 5) with standardized regression weights. Note: +mood—positive mood, −mood—negative mood, relhumor—religion and humor.

Amotivation was removed from the model re-specification due to its multicollinearity with several other variables and high standardized residual covariance. The variable’s elimination was also discussed with a sports psychologist in order to reach consensus. However, because the fit indices for the re-specified model (Model 4) remained outside the acceptable range, the model was further re-specified by adding new paths or correlations, based on the recommendations of modification indices (MI), and after discussions among the researchers. Model 5 was then developed and it was found that it fitted the data well (see Table 2, Model 5). There were 41 path relationships that were found to be statistically significant and theoretically important, as shown in Figure 2. Figure 2 shows the results of path relationships between the study variables of coping, mood, exercise behaviors, and mental health.

Based on Model 5, there were several significant indirect relationships between coping skills and mental health, through exercise behaviors (*p* < 0.05). There were also several significant indirect relationships between mood and mental health, through exercise behaviors (*p* < 0.05).

## 4. Discussion

To accomplish the study’s objective, the path relationship between the study variables of mood, exercise behavior, coping, and mental health was developed using path analysis. In the current study, participants with a negative mood demonstrated less identified regulation. Respondents who were depressed could not be motivated by the significance of a behavior or accept it as a self-regulation. Additionally, the results indicated that those with negative moods were unable to fully integrate exercise into their lives, and thus had a lower level of integrated motivation. These findings were consistent with Annesi and Gorjala [29], who established that long-term negative mood was associated with self-regulation deficits and decreased self-efficacy.

Positive mood was associated with negative intrinsic regulation, whereas negative mood was associated with positive intrinsic regulation. Those with a positive mood were less motivated by intrinsic regulation during the COVID-19 pandemic. These findings contrast with those of Quartiroli et al. [15], who discovered a correlation between positive mood and physical activity. Exercise, in theory, promotes a positive mood [15,31]. Additionally, it is possible that individuals who scored higher for negative mood have a greater capacity for intrinsic regulation. According to experts, when people with low moods use exercise as a coping mechanism, their moods can improve. Similarly, Miller et al. [32] discovered a significant positive correlation between exercise-induced mood and exercise self-efficacy. However, Li et al. [33] discovered that exercise dependence has a detrimental effect on psychological health; in their study of 1601 college students, students who were exercise dependent had higher depression and anxiety scores, which had a negative effect on their moods.

The present study’s findings indicated that mood had an effect on mental health, which is consistent with Ogden and Mtandabari [34], who asserted that mood and mental health had a positive relationship. A high level of positive mood, paired with a low level of negative mood is believed to be associated with a better mental health condition. According to the findings of this study, respondents who reported a positive mood were more likely to report lower levels of anxiety. This is corroborated by the findings of a previous study [34], which indicated that those with a more positive mood experienced less anxiety. There was a significant negative relationship between the respondents’ positive mood and stress. This finding was consistent with the findings of Charles et al. [35], which indicated that respondents with a positive mood experienced higher levels of stress.

According to Ogden and Mtandabari [34], there was a decline in mood and an increase in depression and anxiety. This study corroborates the current study’s finding that those with a negative mood experienced higher levels of depression and anxiety. Respondents with negative moods reported higher levels of stress, which is consistent with previous research [35]. During the COVID-19 pandemic, school and university closures, work-from-home policies, and restrictions on recreational activities were implemented to reduce the risk of virus transmission. However, these measures may have exacerbated people’s negative moods and easily resulted in depression, anxiety, and stress. This was because many people had lost their jobs, relationships, and opportunities—or even their simple pleasures, such as physical contact with family and friends, travel to nearby areas, and interaction with colleagues [36]. They were unable to alleviate their negative emotions through social interaction, which resulted in a depressed mood and increased depression, anxiety, and stress levels.

According to Psederska et al. [37], there was a significant negative correlation between positive mood and depression and anxiety, as well as a positive correlation between negative mood and depression and anxiety. Apart from having a direct effect on mental health, mood has also had an indirect effect on mental health via exercise behavior. Positivity was shown to have increased physical activity and improved the mental health status of Malaysians during the COVID-19 pandemic. External regulation was found to have a significant positive relationship with depression, anxiety, and stress in this study. Externally regulated individuals reported higher levels of depression, anxiety, and stress. According to Vancampfort et al. [38], external motivations to exercise could result in depression. This was because the presence of contingencies, such as the COVID-19 pandemic, mediated these external behaviors, and when the contingencies were removed, the exercise behaviors deteriorated [39].

Additionally, introjected regulation was positively associated with stress. Individuals with introjected regulation had significantly higher stress scores. They exercised out of guilt. Vancampfort et al. [38] found that when people were under pressure to think, feel, or act in particular ways (for example, when they were experiencing guilt or seeking public recognition), they were less likely to engage in physical activity. When people engage in less physical activity, they experience an increase in depression, anxiety, and stress. Introjected regulation, on the other hand, had a significant negative relationship with depression. The findings indicated that those with introjected regulation had lower levels of depression. This finding differs from that of Vancampfort et al. [38]. While feeling guilty about exercising may result in elevated levels of depression, our findings indicated that introjected regulation and depression were inversely related. It is possible that although they exercised initially out of guilt, over time, regular exercise could help them overcome depression. It is also possible that abstaining from physical activity out of guilt may also help to alleviate their depression. Raglin [40] supports this by stating that excessive exercise can result in a decline in physical health.

Furthermore, the identified regulation was associated with depression and anxiety in a significant way, both positively and negatively, respectively. The findings from the current study indicated that those who exercised with identified regulation had lower depression and anxiety scores, as a result of their ability to value the benefit of exercise. This result suggests that exercise can help to reduce depression and anxiety. These findings corroborate those of Khanzada et al. [41], who discovered that regular exercise was associated with a significant reduction in the frequency of depression and anxiety in a cross-sectional study of 269 individuals. Additionally, in this study, stress was found to have a significant positive relationship with identified regulation. Those who exercise under identified regulation do so in order to achieve personally desired outcomes. In this instance, those who used identified regulation scored higher on the stress scale. This can be explained by the fact that individuals who do not engage in physical activities are unable to appreciate the value of the positive outcomes associated with them [38], resulting in an increase in stress. According to Blevins et al. [42], physical activity has been shown to be an effective method of mood regulation. The study discovered that the participants’ use of adaptive coping strategies and autonomous motivation (intrinsic regulation) increased over time. Our findings indicated a significant positive relationship between approach coping and external regulation, introjected regulation, identified regulation, integrated regulation, and intrinsic regulation. Among all the various exercise regulations, intrinsic regulation, along with approach coping, had the greatest effect, which was consistent with Dawson and Golijani-Moghaddam [43].

Integrated regulation had a positive relationship with approach coping, avoidant coping, religion, and humor. Those who engaged in approach coping demonstrated a higher level of integrated regulation. Those who used approach coping, avoidant coping, religion, or humor during the COVID-19 pandemic tended to believe that they needed to exercise because they desired to exercise. They believed exercise was a natural part of their lives, which allowed them to exercise in a guilt-free and shame-free way. As a result, this trend was more pronounced for those who used approach coping, which was consistent with Ma et al.’s [44] findings that the publics’ coping styles are associated with exercise behavior. Thus, the more negative a coping style is, the lower the score for active exercise behavior. Approach coping, religion, and humor all had significant beneficial effects on intrinsic regulation. Individuals with approach coping abilities performed better on intrinsic regulation. When compared to religion and humor, the effect is seen to be strong, indicating that those who use approach coping skills exercise because they enjoy it and that their motivation comes from the joy of exercise, not from the promise of a prize or any other influence. These findings are corroborated by Blevins et al. [42], who found that changes in intrinsic regulation (autonomous motivation) were positively and significantly associated with adaptive coping. Additionally, according to Kim and McKenzie [45], exercising during leisure time promotes effective coping by improving coping skills.

In contrast, avoidant coping was associated with a significant positive relationship with depression, anxiety, and stress. Our findings corroborate those of Yan et al. [46], who found that people who used more negative coping skills were more likely to experience stress, putting them in a “high-risk” category for mental illness. Dawson and Golijani-Moghaddam [43], established that individuals who engaged in avoidant coping behaviors were more likely to be distressed and to have poor mental health. According to MacIntyre et al. [47], fewer approach coping skills and a greater reliance on avoidant coping skills were associated with more negative outcomes. Additionally, some studies [9,48] have discovered that those who used active avoidance coping skills were unable to resolve problems during the COVID-19 lockdown, potentially increasing the risk of symptoms. By using avoidance behavior as a coping mechanism, individuals learn to avoid unpleasant situations, resulting in the aggravation of psychological problems and decreased optimistic thinking.

Finally, religion and humor were negatively associated with depression and anxiety. Garcia et al. [49] found that humor had a positive correlation with well-being and a negative correlation with perceived stress. Similarly, Dulmus and Hilarski [50] discovered that some people used their religious beliefs to overcome depression, anxiety, and stress. Adults reported coping with social isolation and distance through humor and religious activities in Wildman et al.’s [51] study. Apart from directly affecting mental health, these coping strategies also had an indirect effect on mental health via exercise behavior. Adapting positive coping skills was found to increase exercise behavior and thus improve mental health among Malaysians during the COVID-19 pandemic.

### Strengths, Limitations, and Future Directions

This is the first study to examine the causal relationships between mood, physical activity, coping, and mental health in Malaysians during the COVID-19 pandemic. As a result, the study’s findings could serve as a baseline for future research or could be used by healthcare providers to develop a plan for future crises, such as the establishment of a hotline for psychological assistance. Second, path analysis necessitates a large sample size in order to estimate parameters precisely. The current study recruited a sufficient number of respondents to address these issues. Additionally, this study sheds light on the variables’ direct and indirect effects on mental health. Furthermore, the instruments used in this study (questionnaires) were valid and reliable. All questionnaires were available in two languages: Malay and English, which enabled respondents to comprehend and accurately answer the questions.

There were a number of limitations to this study. The first was related to the sampling technique used. Malaysia was chosen as the study’s target country. Due to the geographical area being too large and diverse to obtain the sample size required for this study, respondents were recruited via snowball sampling, rather than random sampling. This was one of the study’s limitations in terms of generalizability. As a result, it cannot be said to represent the entire population of Malaysia. Second, respondents were recruited via an online Google Forms survey. Online surveys require access to the internet, and respondents must be fluent in Malay and English, which appeared to exclude non-English or Malay speakers and those from lower socioeconomic backgrounds from participation. Additionally, users who were unfamiliar with technology, such as the elderly, were underrepresented. As a result, respondents’ sociodemographic characteristics revealed an overrepresentation of university-educated individuals, which may limit the generalizability of the findings. If time and resources permit, future studies should recruit respondents equally by the state of residence, gender, ethnic origin, and occupation to ensure the study’s generalizability. A multistage random sampling method is proposed for future studies to ensure sample equality across Malaysia’s states.

## 5. Conclusions

This study elucidated the relationships between mood, coping, exercise behavior, and mental health in Malaysians. According to the study’s final model, a total of 41 paths were supported and were statistically significant. In general, the study variables’ path relationships were established. It was demonstrated that the variables of mood, coping and exercise behavior had both direct and indirect effects on mental health. It was shown that mental health status can be indirectly improved by appropriate coping skills, through exercise behaviors, and that mood can also indirectly affect mental health status, through exercise behaviors.

## Figures and Tables

**Table 1:** Sociodemographic characteristics of respondents, (*n* = 842).

Characteristic	Frequency (*n*)	Percentage (%)	Mean (SD)	Median (IQR)
Gender				
Male	202	(24.0)		
Female	640	(76.0)		
Age (years)				22 (6) *
Ethnicity:				
Malay	440	(52.3)		
Chinese	308	(36.6)		
Indian	48	(5.7)		
Others	46	(5.5)		
Occupation:				
Employed with wages (full time)/ self-employed	262	(31.1)		
A housewife	7	(0.8)		
A student	561	(66.6)		
Unable to work	12	(1.4)		
Household income:				
I don’t have an income at the moment	539	(64.0)		
Below RM 4360	147	(17.5)		
Between RM 4360 to RM 9616	109	(12.9)		
Above RM 9616	47	(5.6)		
Education:				
No formal education/Primary education/ Secondary education	24	(2.9)		
Undergraduate degree	656	(77.9)		
Postgraduate degree	145	(17.2)		
Others	17	(2.0)		
Exercise frequency:				
Never	85	(10.1)		
Once in a while	325	(38.6)		
Once a week	107	(12.7)		
Two times a week	88	(10.5)		
Three times a week	86	(10.2)		
Four times a week	35	(4.2)		
Five times a week	29	(3.4)		
Six times a week	17	(2.0)		
Every day	70	(8.3)		
Exercise sessions (minutes per sessions):				
˂10 min	234	(27.8)		
≥10 min	608	(72.2)		
Have you tested positive for COVID-19:				
Yes	5	(0.6)		
No	837	(99.4)		

Note. * data are skewed; SD = standard deviation, IQR = interquartile range.

**Table 2:** Summary of model fit indices for all the models (Models 1–5).

Model	CFI	TLI	SRMR	RMSEA (90%CI)	RMSEA *p*-Value
Model 1 (Initial)	0.936	0.753	0.005	0.317 (0.305, 0.330)	<0.001
Model 2	0.933	0.783	0.005	0.297 (0.286, 0.309)	<0.001
Model 3	0.872	0.666	0.018	0.369 (0.359, 0.379)	<0.001
Model 4	0.848	0.576	0.277	0.416 (0.405, 0.426)	<0.001
Model 5 (Final)	0.998	0.988	0.001	0.072 (0.056, 0.088)	0.012

Note. CFI—comparative fit index, TLI—Tucker–Lewis index, SRMR—standardized root mean square residual, RMSEA—root mean square error of approximation, CI—confidence interval.

## Data Availability

The data that supported the findings of this study are available from the corresponding author, K.Y.C. and G.K., upon reasonable request.
